# Cholecalciferol vs. Small Doses of Alfacalcidol vs. Placebo in Chronic Kidney Disease Patients on Hemodialysis: A Randomized Parallel Group Study

**DOI:** 10.3389/fmed.2021.781191

**Published:** 2022-01-21

**Authors:** Joanna Matuszkiewicz-Rowińska, Paweł Kulicki, Paweł Zebrowski, Wiesław Klatko, Antoni Sokalski, Stanisław Niemczyk, Magdalena Wypych-Birecka, Jolanta Małyszko

**Affiliations:** ^1^Department of Nephrology, Dialysis and Internal Medicine, Medical University of Warsaw, Warsaw, Poland; ^2^Nephrology Department, Regional Specialty Hospital, Ciechanów, Poland; ^3^Dialysis Unit and Nephrology Department, Regional Specialty Hospital, Radom, Poland; ^4^Department of Internal Medicine Nephrology and Dialysis, Military Medical Institute of Warsaw, Warsaw, Poland; ^5^Diaverum Dialysis Center, Warszawa, Poland

**Keywords:** vitamin D, 1, 25(OH)_2_D, hemodialysis, alfacalcidol, cholecalciferol

## Abstract

**Background:**

The ability of extrarenal tissues to convert 25(OH)D (calcidiol) into 1,25(OH)2D (calcitriol) and dependence of the conversion on substrate levels provide the rationale for supplementing vitamin D in dialysis patients who usually have severe depletion of both: 25(OH)D and 1,25(OH)2D. The primary aim of the study was to compare effects of small doses of cholecalciferol (12,000 IU/week) with frequently used in Europe, small doses of alfacalcidol (1.5 μg/week) or placebo, given for 12 weeks, on serum 1,25(OH)_2_D in hemodialysis patients with 25(OH)D deficiency. Secondary outcomes were changes in serum calcium, phosphate, 25(OH)D, parathyroid hormone (PTH), fibroblast growth factor 23 (FGF23) and sclerostin during the treatment.

**Methods:**

This was a prospective, randomized, partly double-blind (cholecalciferol vs. placebo) study. Out of 522 patients dialyzed in 5 centers in the Mazovian Province, 93 gave informed consent and met the inclusion criteria: any vitamin D metabolites and calcimimetics naïve; no history of liver or intestinal disease; serum 25(OH)D <20 ng/ml, iPTH <1,000 –>110 pg/ml, calcium <10.2, and phosphate <6.8 mg/dl. The subjects were stratified by serum iPTH, then randomized into 3 groups according to the treatment.

**Results:**

To our knowledge, this is the first study comparing head-to-head these drugs in the hemodialysis population. There were no significant differences between the groups at baseline. 81 patients completed the study. Cholecalciferol normalized serum 25(OH)D, with a mean rise from 12.9 ± 6.7 to 31.3 ± 10.1 ng/ml (*p* < 0.0001). This was accompanied by a marked increase of 1,25(OH)_2_D from 13.8 ± 9.3 to 25.1 ± 14.2 pmol/l (*p* < 0.0001). A rise in serum 1,25(OH)_2_D was also observed in alfacalcidol treated patients, however much smaller (from 13.5 ± 10.1 to 18.5 ± 11.0 pmol/l; *p* = 0.02). Neither cholecalciferol nor alfacalcidol treatment resulted in significant changes in serum PTH and the remaining parameters.

**Conclusions:**

In most patients, treatment with cholecalciferol in a 12,000 IU/week dose permits safe correction of 25(OH)D deficiency and is more effective than 1.5 μg/week dose of alfacalcidol in rising serum 1,25(OH)_2_D. This, together with a lack of influence on circulating iPTH the usefulness of such small alfacalcidol doses in hemodialysis patients is debatable.

## Introduction

According to current knowledge, vitamin D regulates the function of many organs and systems, not only mineral and bone metabolism. Moreover, it has been postulated that its deficiency may be associated with an increased risk for nearly all major human diseases. We know now that both 1-alpha-hydroxylase (CYP27B1) and vitamin D receptor (VDR) are present in almost every human tissue and that vitamin D may exert its actions *via* two general ways. These are: (1) the endocrine way with 1,25(OH)2D (calcitriol) as a hormone produced in kidneys, and (2) paracrine, autocrine and intracrine ways, in which its precursor −25(OH)D (calcidiol) is converted locally by CYP27B1 to 1,25(OH)2D in the target cell, which activates the VDR and downstream gene expression in the same or a neighboring, VDR-expressing cell (1). Moreover, a number of studies documented that this localized, tissue-specific conversion is a key determinant of many physiological processes and that it is substrate-dependent (2–5). The recognition of the ability of extrarenal tissues to produce calcitriol and the suggestions that many of the significant biological consequences of dysregulated vitamin D balance may be associated with changes in the extracellular concentration of substrate 25(OH)D together with the fact of severe deficiency of both, 1,25(OH)2D and 25(OH)D, in patients on long-term dialysis therapy provided a rationale to the study. In addition, since oral alfacalcidol is a popular VDR activator analog in many countries, in some cases given in a small dose (6–9), we decided to examine if this therapy has any advantage over nutritional vitamin D supplementation.

The study's primary outcome was the effect of 12-week therapy of cholecalciferol compared with low-dose alfacalcidol or placebo on serum 1,25(OH)2D in vitamin D naive hemodialysis patients with 25(OH)D deficiency. Secondary outcomes were changes in selected circulating markers of mineral metabolism during the treatment.

## Materials and Methods

**Figure 1:**
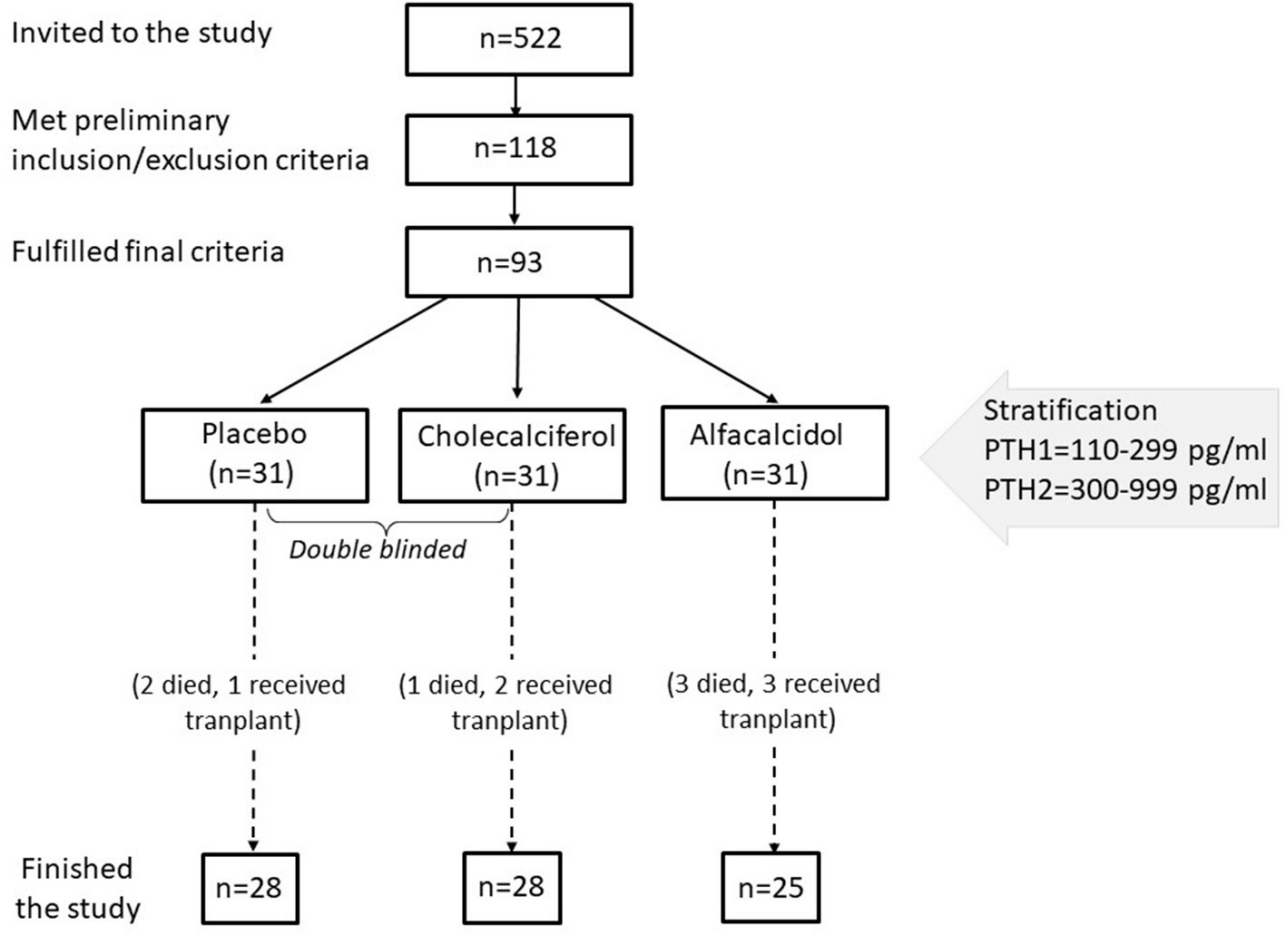
The flow diagram of patients selection.

All adult patients (522 in total) hemodialyzed in 5 cooperating centers in the Mazovian Province had been analyzed. Out of them, 118 patients who signed written informed consent, were at least 3 months and fulfilled none of the exclusion criteria were invited to the first part of the study. The exclusion criteria were: the treatment with any vitamin D metabolites or calcimimetics in the last 6 months, the history of the parathyroid surgery, cancer disease, or severe general condition. Then serum 25(OH)D was measured in the central lab, and 93 patients with levels below 20 ng/ml and fulfilling the other criteria entered a final phase of the study. These additional criteria were: serum parathyroid hormone (PTH) <1,000 and >110 pg/ml, serum calcium ≤ 10.2 mg/dl, serum phosphate <6.8 mg/dl, and written consent for this part of the study. The study was partly double-blind (for cholecalciferol vs. placebo comparison). Enrolled subjects were stratified by serum PTH and randomly assigned in 1:1:1 ratio to oral cholecalciferol (two capsules á 2,000 IU), alfacalcidol (two capsules á 0.25 μg), or placebo (two capsules), taken three times a week, during hemodialysis for 3 months. The study protocol was approved by the University of Warsaw Ethical Committee (KB/266/2012). All patients provided their written informed consent to participate in this study. The flow diagram of patients selection is presented in Figure 1.

Cholecalciferol and placebo capsules (containing organoleptically matched triglyceride oil) were identical and were kindly supplied by Oleofarm under the label D Vitum Forte; GlaxoSmithKline Pharmaceuticals produced alfacalcidol capsules. All drugs were given by a nurse. The study team was unaware of treatment allocation. A qualified person from an independent institution did the randomized labeling, packaging, and final release.

The observation period was 13 weeks. The dosage of studied drugs remained unchanged throughout the study. At the same time, previous treatment was continued in all patients. The subjects were asked to refrain from taking any vitamin supplements for the duration of the trial. Hemodialyses were performed using single-use polysulphone or polyamide dialyzers and bicarbonate-based dialysis fluid. The duration of the procedures ranged from 3.5 to 5.5 h., with dialysis dosage being modified monthly so that Kt/V for urea was ≥1.2. During the study, local laboratories in participating centers determined serum total calcium and phosphate at baseline, after two, five, nine, and 13 weeks. The measurements of screening serum 25(OH)D and intact (i)PTH to determine study eligibility, as well as serum 1,25(OH)2D, 25(OH)D, iPTH, fibroblast growth factor 23 (FGF23), and sclerostin before and after the study, were done centrally. Blood for these analyses was collected from fasting patients before the dialysis; after centrifugation of samples, serum was immediately frozen at ca. −70°C and sent in dry ice to the central laboratory at the Medical University of Warsaw.

Serum concentrations of iPTH and 25(OH)D were measured using highly sensitive electrochemiluminescence immunoassays on an Elecsys 2010 automatic analyzer (Roche Diagnostics GmbH, Mannheim, Germany). The reference manufacturer's data were 15–65 pg/mL for iPTH and 11.1–42.9 ng/mL (27.7–107 nmol/L) for 25(OH)D. Serum 1,25(OH)2D was measured using a complete, manual assay system cat. No. AC-62F1 (Immunodiagnostic Systems, Frankfurt, Germany) according to the manufacturer's protocol. The system utilizes immunoextraction of 1,25(OH)2D from serum followed by enzyme immunoassay and is more specific toward 1,25(OH)2D3 (100%) than toward 1,25(OH)2D2 (39%). The reference manufacturer's data were 39–193 pmol/L (*n* = 120) for healthy adults and <6–22 pmol/L (*n* = 24) for end-stage renal disease patients. Serum FGF-23 was determined using Human FGF-23 ELISA Kit (cat. number EZHFGF-23-32K) purchased from Millipore (USA), following the manufacturer's instructions. Millipore Human FGF-23 ELISA Kit employs the quantitative sandwich enzyme immunoassay technique. Intra-assay and interassay coefficients of variation were 7.2 and 5.3%, respectively. FGF-23 levels were expressed as pg/ml. Serum sclerostin was measured with a quantitative sandwich ELISA (Sclerostin ELISA, Biomedica, Vienna, Austria) according to the standard protocol provided by the manufacturer. Intra- and interassay coefficients of variation were <7 and <10%, respectively. The reference manufacturer's data were 10.9–28.7 (median 14.3) pmol/l.

Statistical analysis. The results are presented as mean with one standard deviation for normally distributed variables or median and range for non-normally distributed variables as tested by the Lillefors test. A *P* < 0.05 was considered as significant. For statistical significance assessment *T*-Test, One-Way Anova, Wilcoxon, and Mann-Whitney tests were used accordingly. All calculations were performed using STATISTICA software package (version 13), StatSoft Poland.

## Results

The study was started in December. Among 118 patients who fulfilled the preliminary criteria, 116 (98%) had serum 25(OH)D levels below 30 ng/ml and 97 (82%)—below 20 ng/ml. Out of them, 93 patients fulfilled the final criteria and were randomized to the treatment groups. The final analysis included 81 patients, 49 men, and 32 women, who completed the 13-week observation period. Their clinical and biochemical data are presented in Table 1. There were no significant differences between the study groups. There was a significant correlation between serum 25(OH)D and the declared amount of time spent outside by patients (Spearman correlation = 0.411, *p* < 0.001). Serum 25(OH)D correlated with a duration of dialysis treatment (r = −0.272, *p* = 0.014), residual diuresis (r = 0.289, *p* < 0.01), serum phosphate (r = −0.393, *p* < 0.001), FGF23 (r = −0.295, *p* = 0.008), and sclerostin (r = −0.260, *p* = 0.019). Serum sclerostin correlated also with age (r = 0.356, *p* = 0.001) and a duration of dialysis treatment (r = 0.402, *p* < 0.001); and it was significantly higher in men than in women (*p* < 0.03). The study showed a very high dispersion of serum FGF23 concentrations in the population, from 4.71 to 27 612 pg/ml, with the median 379 pg/ml. Apart of serum 25(OH)D, serum FGF23 correlated with serum phosphate (r = 0.549, *p* < 0.001) and calcium (r = 0.328, *p* = 0.003).

**Table 1:** The clinical and biochemical parameters of the 81 patients who finally took part in the study.

**Parameter**	**Mean (range)**	**Median**	**Lower quartile**	**Upper quartile**
Age (years)	67 ± 13 (25–91)	67	59	77
Dialysis vintage (months)	51 ± 54 (7–276)	36	16	65
Time spent outside (hours/d)	3.2 ± 1.1 (1.0–5.0)	3.0	2.0	4.0
25(OH)D (ng/dl)	13.4 ± 6.72 (3.0–29.4)	11.9	8.46	17.5
1,25(OH)_2_D (pmol/l)	12.9 ± 9.08 (2.61–38.7)	8.84	6.94	16.1
iPTH (pg/ml)	347 ± 189 (112–904)	300	212	410
FGF23 (pg/ml)	2 823 ± 5 647 (4.71–27 612)	379	90.7	2 633
Sclerostin (pmol/l)	89.2 ± 46.7 (5.11–284)	81.9	58.3	104
Total calcium (mg/dl)	8.73 ± 0.65 (7.10–10.2)	8.76	8.3	9.2
Phosphate (mg/dl)	4.97 ± 1.14 (2.50–6.81)	5.19	4.2	5.6

The 13-week treatment with cholecalciferol significantly increased serum 25(OH)D in studied patients (Table 2); in all of them except one, the values exceeded 20 ng/ml, and in 60% of the patients −30 ng/ml. This was accompanied by a marked (*p* < 0.0001) increase of serum 1,25(OH)2D (Figure 2). A rise in serum 1,25(OH)2D was also observed in alfacalcidol-treated patients, however much smaller (*p* < 0.02). As expected, no changes were observed in the placebo group. No significant changes and differences in serum calcium, phosphate as well as no significant effects of the tested regimens on serum iPTH, FGF23, and sclerostin concentrations were observed (Table 2). In two patients from the cholesterol group, temporary mild serum phosphate increases were observed, necessitating non-calcemic phosphate binders administration.

**Table 2:** Changes in biochemical parameters during the treatment in three studied groups.

	**Cholecalciferol *Mean ± SD; median (range)***	**Alfacalcidol *Mean ± SD;* *median (range)***	**Placebo *Mean ± SD; median (range)***
	**25(OH)D (ng/ml)**		
Before	12.9 ± 6.7; 10.7^*^ (3.13–28.8)	11.7 ± 7.20; 10.1 (3.29–29.2)	15.4 ± 5.97; 14.8 (8.02–29.4)
After	31.3 ± 10.1; 32.0 (11.8–52.3)	10.1 ± 5.76; 11.0 (3.00–22.2)	13.1 ± 6.37; 11.5 (3.00–26.6)
	**1,25(OH)2D (pmol/l)**		
Before	13.8 ± 9.27; 9.52^*^ (4.95–38.7)	13.5 ± 10.1; 8.52^**^ (2.61–37.0)	11.5 ± 7.95; 8.59 (4.06–32.7)
After	25.1 ± 14.23; 20.2 (6.31–70.6)	18.5 ± 11.0; 16.4 (3.09–48.8)	14.8 ± 10.3; 12.7 (3.13–53.1)
	**Total calcium (mg/dl)**		
Before	8.72 ± 0.74; 8.75 (7.45–10.2)	8.69 ± 0.50; 8.70 (7.16–9.36)	8.78 ± 0.68; 8.89 (7.10–9.87)
After	8.82 ± 0.72; 8.81 (6.30–10.3)	8.78 ± 0.61; 8.70 (7.40–10.0)	8.67 ± 0.97; 8.84 (5.6–10.3)
	**Phosphate (mg/dl)**		
Before	4.81 ± 1.08; 4.61 (2.80–6.60)	5.12 ± 1.20; 5.42 (2.50–7.26)	4.99 ± 1.16; 5.25 (2.67–6.80)
After	5.88 ± 1.97; 5.54 (2.64–12.9)	5.30 ± 1.51; 5.00 (2.70–8.61)	5.17 ± 1.42; 5.37 (2.58–7.47)
	**iPTH (pg/ml)**		
Before	333 ± 187; 293 (125–842)	355 ± 205; 320 (112–904)	354 ± 183; 305 (134–823)
After	417 ± 304; 327 (128–1,469)	311 ± 216; 307 (12.0–860)	425 ± 283; 339 (68.5–1,317)
	**FGF23 (pg/ml)**		
Before	2,130 ± 4,726; 363 (23.8–23 656)	3,373 ± 6,742; 150 (4.7–26 382)	3,044 ± 5,607; 679 (21.4–27 612)
After	2,136 ± 3,153; 882 (24.6–12 880)	3,257 ± 5,426; 380 (10.1–18 860)	3,322 ± 5,524; 626 (6.28–20 788)
	**Sclerostin (pmol/l)**		
Before	83.7 ± 52.5; 80.8 (5.09–284)	93.7 ± 50.6; 78.6 (29.6–202)	90.5 ± 37.1; 87.7 (28.8–184)
After	73.4 ± 34.4; 63.9 (29.1–192)	77.8 ± 40.7; 69.2 (30.9–182)	68.5 ± 32.6; 67.2 (12.4–161)

* *p <0.0001*,

** *p <0.02*.

## Discussion

The rapidly aging dialysis population with a high burden of comorbid illnesses, insufficiently exposed to the sun, or affected by malnutrition, is particularly vulnerable to bone fractures due to profound disturbances in mineral metabolism. From the same reasons together with defective cutaneous cholecalciferol synthesis and the effects of a variety of medications that prevent its intestinal absorption or interfere with its metabolism (10–12), calcidiol deficiency is a common finding in this population, ranging from 38 to 95%, depending on the definition, geographic latitude, and season of the year (13–18).

Poland is a big European country that stretches between 49 and 54°latitude North, with a climate similar to Germany and northern France. In a large multicenter Polish study, aimed to prospectively assess 25(OH)D seasonal fluctuations in a cohort of 210 vitamin D naive hemodialysis patients, in wintertime, 82% of subjects had calcidiol deficiency (<20 ng/ml), with one-third of them being severely deficient (<10 ng/ml) (19). Our study showed similar results with only 2% of patients having serum 25(OH)D ≥30 ng/ml, and 82%—below 20 ng/ml, which is considered to be a deficiency and is associated with unfavorable skeletal outcomes, including fractures and bone loss (20, 21). It should be underlined that the patients with severe general conditions were excluded from the study.

Despite the alarming results of the studies evaluating 25(OH)D deficiency in dialysis populations worldwide, the current nephrology societies leave us without a clear guideline on that issue. In 2009 and 2017, KDIGO proposed, with a low quality of evidence, measuring 25(OH)D serum and treating its deficiency as in the general population, however, without any suggestion concerning the dosing and the target threshold (22, 23). With such a weak and imprecise recommendation, many patients undergoing dialysis remain without native vitamin D supplementation. During the preliminary selection to our study, performed among 522 patients in 5 centers, native vitamin D was taken by less than half of them and often as a part of multivitamin preparations. The main argument for neglecting native vitamin D supplementation in patients on dialysis has been the fact of a weak 1,25(OH)2D production by severely damaged kidneys. However, the discoveries of the last decades showing a presence of high extrarenal synthesis of calcitriol point anew to the importance of native vitamin D supplementation in those with end-stage kidney disease undergoing dialysis (24).

Based on the available studies as a minimal dose of cholecalciferol which could effectively replenish 25(OH)D deficits in studied subjects, we assumed 12,000 IU per week, divided into three single doses given during every hemodialysis. The treatment period was short; however, this dose of cholecalciferol normalized (≥30 ng/ml) serum 25(OH)D in half of the group, and in the remaining patients, except one, the serum levels increased above 20 ng/ml. A cut-off level of 20 ng/ml has been recommended as a minimal target by different societies and expert bodies, including the Institute of Medicine (IOM, USA), according to which this level covers the requirements of at least 97.5% of the population (21, 25, 26). However, it remains to be verified in clinical studies if these serum 25(OH)D concentrations are sufficient to fully capture the effect of the localized, tissue-specific conversion to 1,25(OH)2D in ESRD with their specific mineral-bone and other uremia-related disorders. The observed by us a marked (*p* < 0.0001) increase of serum 1,25(OH)2D concentrations confirms the significance of that effect and is consistent with the findings of the other authors (27–30). The treatment was safe; there were no episodes of hypercalcemia. In none of the patients serum 25(OH)D concentrations exceed 60 ng/ml.

As in the other randomized studies, no significant changes in serum PTH concentration were found during the treatment (27, 29–34). In patients on dialysis, the pharmacological doses of VDR activators are necessary for that purpose (35). The reduction of PTH secretion has been for many years the primary goal of calcitriol therapy in many patients at the price of positive calcium balance with substantial tissue calcifications. The introduction of calcimimetics allowed at least partly to solve that problem. However, as we now know, the role of vitamin D is not confined to parathyroid suppression and has many other essential actions in the bone and other tissues. Therefore, the normalization of serum 25(OH)D and the achievement and maintenance of higher, although still below the normal range 1,25(OH)2D levels without a significant risk of toxicity, seems reasonable.

An important part of our study was to compare the effects of small doses of cholecalciferol with small doses of alfacalcidol on serum 1,25(OH)2D in hemodialysis calcidiol deficient patients. For alfacalcidol, we decided to choose the dose of 0.5 μg thrice a week, which equals the dose of 0.25 μg daily, since it is still given in clinical practice in Europe, although data on its efficacy are scarce if any. The aim was to examine if this therapy has any advantage over nutritional vitamin D supplementation. The treatment with alfacalcidol caused a rise in serum 1,25(OH)2D, however much smaller than in the cholecalciferol group (medians: from 8.59 to 12.7 vs. from 8.52 to 16.4 pmol/l, respectively). Similar observations reported Rajah et al. (36), who examined the biochemical response to alfacalcidol and subsequently the change in response to ergocalciferol in 10 children with rickets.

Our study has several strengths. First, it was a prospective, randomized and parallel design, partly double-blinded study. All drugs were given by a nurse. Besides, the studied subjects were vitamin D naïve, all of the same race, living in similar weather conditions. The limitations are a relatively small number of patients and a short observation time.

These results question the usefulness of alfacalcidol in a dose of 1.5 μg/week in hemodialysis patients as ineffective: it has a weak influence on serum 1,25(OH)2D and no effects on serum PTH concentrations. This practice is debatable, prospective multicenter studies on larger populations might answer the question whether this therapeutic approach is efficient. The low-dose cholecalciferol supplementation raises serum 1,25(OH)2D more effectively, replenishes 25(OH)D stores, and is safe and cheap. Although a low dose of cholecalciferol can increase 1,25(OH)2D, its benefit in dialysis patients remains an open issue.

## Data Availability Statement

The original contributions presented in the study are included in the article/supplementary material, further inquiries can be directed to the corresponding author.

## Ethics Statement

The studies involving human participants were reviewed and approved by University of Warsaw Ethical Committee (KB/266/2012). The patients/participants provided their written informed consent to participate in this study.

## Author Contributions

JM-R and PK designed the study. PK, PZ, WK, AS, SN, and MW-B performed the experiments. PK, JM-R, and JM analyzed the data. JM-R wrote the paper in consultation with JM. All authors contributed to the article and approved the submitted version.

## Conflict of Interest

The authors declare that the research was conducted in the absence of any commercial or financial relationships that could be construed as a potential conflict of interest.

## Publisher's Note

All claims expressed in this article are solely those of the authors and do not necessarily represent those of their affiliated organizations, or those of the publisher, the editors and the reviewers. Any product that may be evaluated in this article, or claim that may be made by its manufacturer, is not guaranteed or endorsed by the publisher.
